# TiC Reinforcement Composite Coating Produced Using Graphite of the Cast Iron by Laser Cladding

**DOI:** 10.3390/ma9100815

**Published:** 2016-09-30

**Authors:** Yanhui Liu, Weicheng Qu, Yu Su

**Affiliations:** School of Materials Engineering, Shanghai University of Engineering Science, Shanghai 201620, China; quwc2015@outlook.com (W.Q.); xlwsy@hotmail.com (Y.S.)

**Keywords:** laser cladding, microstructure, wear resistance, composite coating, cast iron

## Abstract

In this study, a TiC-reinforced composite coating was produced to improve the wear resistance of a pearlite matrix grey iron using a pre-placed Ti powder by laser cladding. Results of scanning electron microscopy (SEM), X-ray diffractometer (XRD), and energy dispersive X-ray spectroscopy (EDS) confirmed that the coating was composed of TiC particles and two kinds of *α*-Fe phase. The fine TiC particles were only a few microns in size and uniformly distributed on the matrix phase in the composite coating. The microstructure characteristic of the composite coating resulted in the microhardness rising to about 1000 HV0.3 (China GB/T 4342-1991) and the wear resistance significantly increased relative to the substrate. In addition, the fine and homogeneous solidification microstructure without graphite phase in the transition zone led to a good metallurgical bonding and transition between the coating and the substrate. It was of great significance for the cast iron to modify the surface and repair surface defects or surface damage.

## 1. Introduction

With the wide application of grey cast iron, longer useful life and better performance, such as thermal fatigue resistance, wear resistance, and corrosion resistance, were expected [1,2,3]. Therefore, many surface engineering technologies have been used on grey cast iron, using both traditional thermal diffusion technologies and modern surface engineering technologies [4,5,6]. Laser surface processing, one kind of surface modifying technology, was also used on grey cast iron to improve the surface properties. For example, Yang et al. [7] investigated the nanomechanical properties and thermal fatigue resistance of grey cast iron processed by laser alloying. They found that the nanohardness of the alloying zone was improved pronouncedly by generating a homogeneous microstructure which has upgraded chemical composition and refined grains. Pang et al. [8] produced striature bionic coupling units by laser surface remelting to improve the wear resistance of grey cast iron guide rails. They found that there was a relationship between weight loss and the area of striature bionic coupling units under dry sliding conditions at room temperature using a homemade linear reciprocating wear testing machine. Jing et al. [9] studied the wear resistance of graphite cast iron brake discs with bionic units processed by laser cladding WC (tungsten carbide) and laser remelting. Their results indicated that the wear resistance of laser cladding WC samples was superior to that of laser remelting ones and their wear resistance was enhanced with the increase of WC content. Advantages of the laser surface process for surface engineering were as follows [10]: (i) high optical quality and high energy density; (ii) unique processing advantages, such as being touch-free and vacuum-free, flexibility (within the fiber) and green processing, high controllability and high efficiency; and (iii) various technical methods, such as laser cladding [11], laser alloying [7], laser remelting [12], laser bionic coupling [13], laser texturing [14,15], laser shock hardening, laser quenching, etc. The above advantages meant laser surface processing had significant developing potential and special industrial competitive force for metal workpieces, as well as the grey cast iron, to improve the surface properties.

The published literature also indicated that the metal matrix composites (MMCs) coating could effectively increase the surface properties of the cast iron [16,17,18]. Especially, in situ synthesis of the reinforcement phases by chemical reactions is attracting more and more attention and research because of its advantages, such as finer particles, natural shape, and good stability of the reinforcement phase, and clean interfaces and good bonding between the reinforcement phase and matrix [19]. In this paper, the pure titanium powder was used to produce a MMC coating on the surface of grey cast iron by laser cladding. The TiC reinforcement phase was synthesized in situ in a laser-melted pool using the pre-placed titanium powder and the lamellar graphite of the grey cast iron. The microstructure and the wear resistance of the coating were studied. This research supplied essential experimental and theoretical bases to improve and repair the surface properties of cast iron parts by laser cladding.

## 2. Experiments

The substrate was a pearlite matrix grey cast iron round sheet with a size of Φ 50 mm × 10 mm. A Ti powder (200 mesh, 99.5% purity) was placed on the clean surface of the substrate. The powder was cold-pressed to eliminate the residual air and to protect the powder from the shielding gas flow. Then, the sample was radiated by a fiber laser system under an argon shielding gas using the following processing parameters: spot size of 5 mm × 5 mm, laser power of 2.2 kW, and scan speed of 7 mm/s. 

The laser cladding samples were cut using an electrospark wire-electrode cutting system and were prepared by standard mechanical grinding and polishing. The samples were etched using 4% nitric acid alcohol solution after mechanical polishing. Phase identification was carried out with a Philips X’Pert PRO X-ray diffractometer (XRD) (PANalytical B.V., Almelo, The Netherlands) using Cu Kα radiation (λ = 1.54 Å). The microstructure morphology and chemical composition were analyzed by scanning electron microscopy (SEM) using a Hitachi S-3400 scanning electron microscope (Tokyo, Japan) with a genesis-type energy dispersive X-ray spectroscopy (EDS) attachment. The microhardness in the cross-section of the coating was investigated by a Vickers hardness tester (Shanghai Taiming Optical Instrument Co. Ltd., Shanghai, China) with a load of 2.94 N and a loading time of 15 s. 

Bruker UMT-2 wear tester (Campbell, CA, USA) was employed to test the wear resistance of the composite coating with the ball-on-disk dry sliding method at room temperature. The ball counterpart was a 440 stainless steel ball with a diameter of 9.5 mm. The wear test lasted for 30 min and the load was 49 N. The eccentric distance was 2 mm and the rotational speed was 100 r/min. Wear mass loss was measured using a AL204-type electronic analytical balance (Mettler Toledo, Shanghai, China) with an accuracy of 0.1 mg. The worn surface was observed by a VHX-600K metallographic microscope (Keyence, Osaka, Japan).

## 3. Results and Discussion

**Figure 1:**
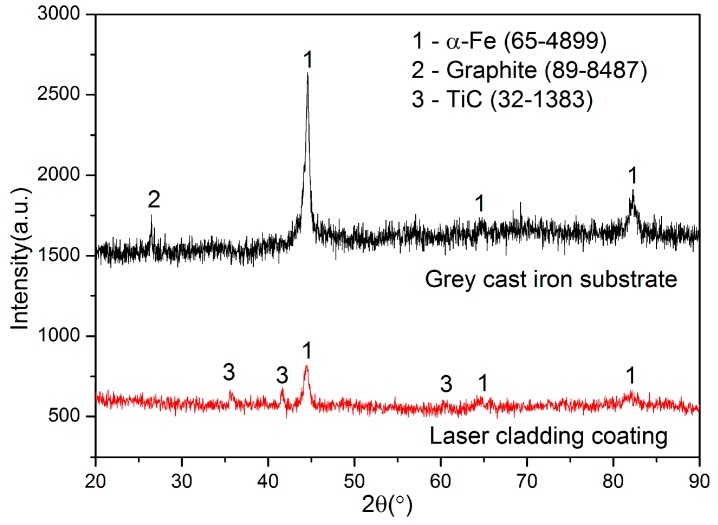
The XRD pattern of the coating and the substrate.

Figure 1 shows the XRD pattern of the laser cladding coating and the substrate. It is clear that the diffraction peaks of graphite phase disappeared and the diffraction peaks of TiC phase appeared after laser cladding. The diffraction peaks of the *α*-Fe phase still exist after laser cladding. According to the chemical composition of the powder and the substrate, it is easy to conclude that TiC was synthesized in the laser-melted pool using the Ti atoms of the pre-placed powder layer and the carbon atoms of the substrate. Obviously, the carbon atoms come from lamellar graphite and combined carbon of the pearlite matrix grey cast iron substrate. 

**Figure 2:**
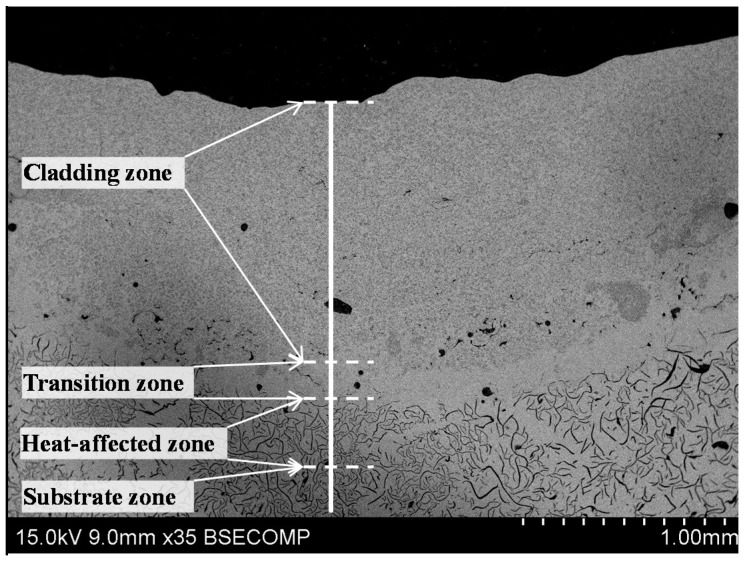
The BSE images of the laser cladding coating on the grey cast iron.

Figure 2 is the backscattered electron-scanning (BSE) image of the laser cladding coating on the grey cast iron. It is clear in Figure 2 that the coating was composed of the cladding zone, the transition zone, and the substrate with the black flake graphite phase. In addition, a heat-affected zone existed in the substrate adjacent to the transition zone. It should be noted that a large amount of the light black particles’ phase, which was about several micrometers in size, was evenly distributed in the cladding zone. Additionally, a few black phases existed in the coating and the substrate in Figure 2. Finally, there were a few black masses in the coating. Most of their shape was round and they were both large and small in size.

**Figure 3:**
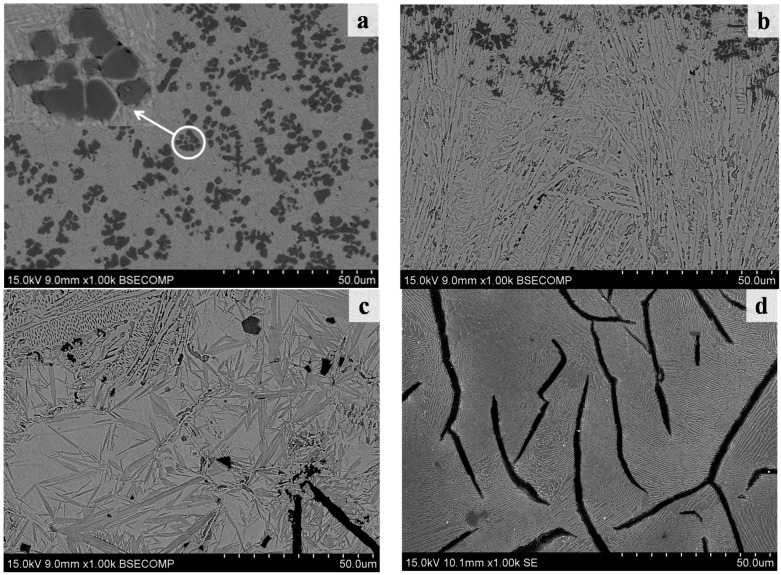
The magnifying BSE images of different zone of the laser cladding coating. (**a**) Laser cladding zone; (**b**) transition zone; (**c**) heat-affected zone; and (**d**) substrate zone.

The magnifying BSE images of different zones in Figure 2 are shown in Figure 3. Figure 3a clearly shows that the light black phase in the cladding zone of the coating was irregularly polygon-shaped and its size was only several micrometers. They were evenly distributed like clusters in the matrix phase. Figure 3b is the SEM image of the transition zone between the cladding zone and the substrate. In the transition zone, the crystalline grain was typical columnar crystal because of the thermolytic characteristics of the laser-molten pool. Figure 3c was the coarse martensite in the heat-affected zone. In addition, the quenching microstructure gradually refined and changed into the normal microstructure (as Figure 3d) of the grey cast iron. It should be noted that one did not find the flake graphite phase in both the cladding zone and the transition zone. Furthermore, one did not find the TiC particle in the transition zone.

**Figure 4:**
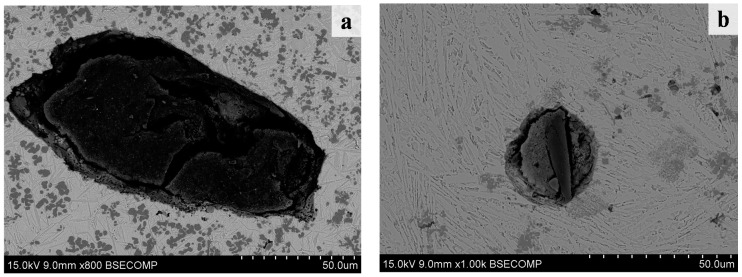
The magnifying BSE images of the black mass in Figure 2. (**a**) Laser cladding zone; and (**b**) transition zone.

The magnifying BSE images of the black round mass in Figure 2 are shown in Figure 4. It could be concluded that the black mass was the graphite phase. Compared with the graphite of Figure 3d, the graphite phase was broken and there were gaps between the matrix and the graphite in Figure 4a,b. Grum [20] and Karamış [21] thought that both hydrodynamic forces and buoyancy forces of the laser molten pool, and the larger size and gradual dissolving speed of graphite particles were the main causes for the graphite particles appearing in the laser cladding coating of the cast iron. In addition, because the thermal expansivity was not matched, the gap appeared between the matrix and the graphite during the fast cooling of the laser molten pool. 

**Figure 5:**
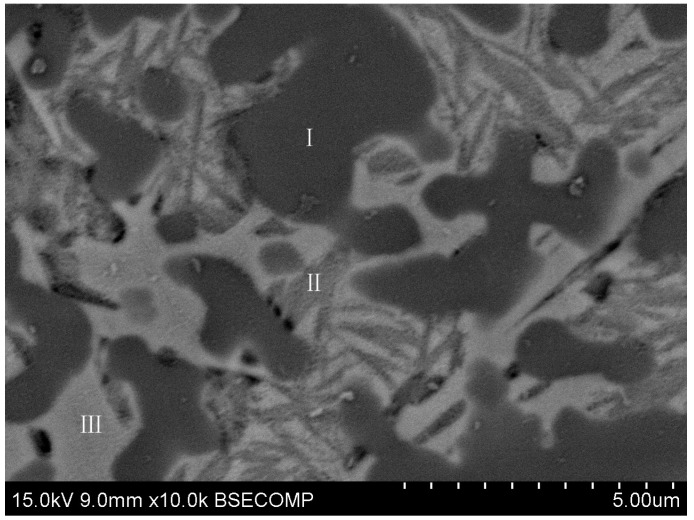
The BSE image of the laser cladding zone. I: Light black particles phase; II: grey matrix phase; and III: grey-white matrix phase.

The further magnifying BSE images of the microstructure of the laser cladding zone in Figure 3a are shown in Figure 5. It was clear that there were three different grey levels, which meant three phases in Figure 5. The results of EDS point analysis at different grey level zones of Figure 5 are listed in Table 1. In the light black particles phase (zone I), Ti at % was 91.16% and Fe at % was 5.09%, and the revised C at % was 3.75%. In the grey matrix phase (zone II), Ti at % was 7.44% and Fe at % was 85.99%, and the revised C at % was 2.80%. In the grey-white matrix phase (zone III), Ti at % was 2.93% and Fe at % was 93.71%, and the revised C at % was 3.36%. Obviously, based on the back-scattered electron imaging principle, chemical composition design, and the XRD results, the black particles phase was the TiC phase, and the matrix was composed of two kinds of *α*-Fe phase. It should be noted that the *α*-Fe phase of zone II in Figure 5 had higher titanium content and was only several hundred nanometers in size. According to the findings of the published literature [22,23], the matrix microstructure was accompanied by some light etching fine Fe_3_C crystal (zone III) and martensite (zone II).

**Figure 6:**
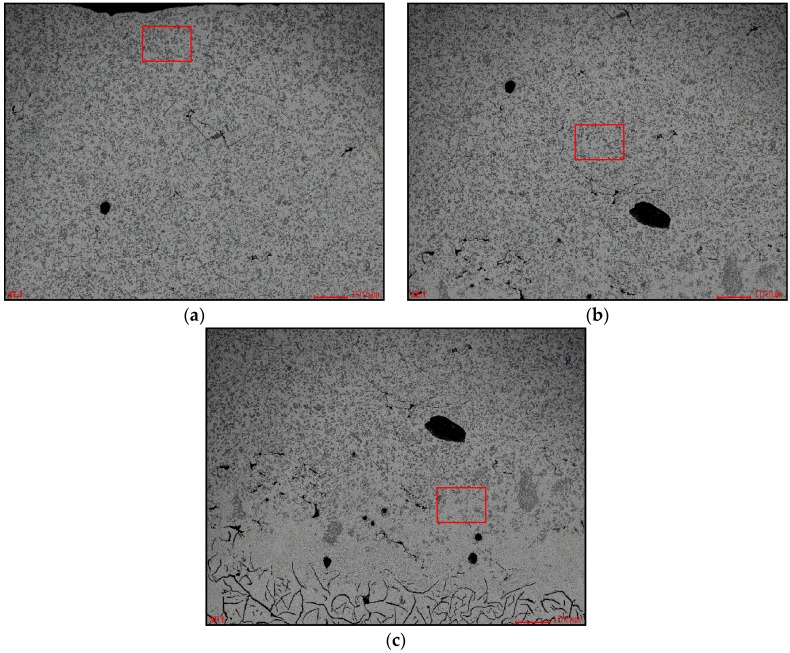
EDS area analysis at the different parts of the laser cladding zone. (**a**) Top of the laser cladding zone; (**b**) middle of the laser cladding zone; and (**c**) bottom of the laser cladding zone. red square: the area for EDS area analysis.

Obviously, the TiC phase was synthesized in the laser molten pool using the titanium atoms of the preset powder layer and the carbon atoms of the molten substrate. Figure 2 and Figure 3a confirmed that the TiC particles were fine and distributed evenly. This meant that there was a driving force to homogenize the titanium atoms and the carbon atoms in the laser molten pool. Theoretical research and experimental results confirmed that Marangoni convection of the laser molten pool was the main driving force [24,25,26,27]. It played a key role for the composition homogenization of the laser molten pool. The results of EDS area analysis at the different parts of the laser cladding zone also confirmed the mass transfer effect of Mrangoni convection. Table 2 listed the results of the EDS area analysis in Figure 6. In Table 2, the Fe content in the top, middle, and bottom of the laser cladding zone was 73.67 at %, 76.17 at %, and 84.73 at %, respectively. This indicated that the Fe was the main element in the laser cladding zone. According to Table 1, the Ti element was mainly found in the TiC particles. Therefore, the Ti content significantly reduced in the bottom of the coating because of the reduction of TiC particles. The characteristic of the microstructure and the distribution of the chemical composition in the laser cladding zone indicated that the convection mass transfer had happened during the laser cladding and led to the synthesis and uniform distribution of TiC.

The microstructure of the transition zone mainly contained the eutectic ledeburite and the plate cementite, which showed the same finding of Abboud [28] and Alabeedi et al. [29]. In addition, the microstructure of the matrix in the laser cladding zone (Figure 3a and Figure 4a) was finer than the microstructure in the transition zone (Figure 3b and Figure 4b). This should be ascribed to the effect of Marangoni convection. 

**Figure 7:**
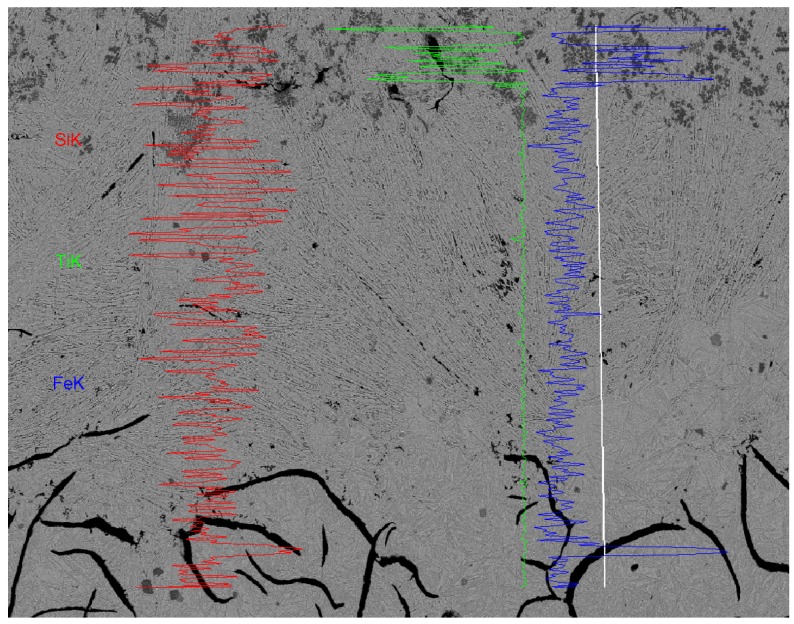
The results and the diagram of the EDS line analysis at the transition zone.

In the transition zone both the TiC particles and the flake graphite phase were not found. The lack of the TiC phase means that the titanium atoms were not diffused into the transition zone. In other words, although the transition zone had been molten, it did not take part in Marangoni convection of the laser molten pool. The disappeared graphite could be ascribed to a rise into the laser cladding zone because of density differences or dissolving into the transition zone. In addition, the investigation of the chemical composition of the transition zone by EDS line analysis in Figure 7 also confirmed that there was almost no Ti in the transition zone, and the distribution of Fe and Si were stable. The characteristics of the microstructure and the chemical composition indicated that the transition zone had melted and solidified during the laser cladding, but it was not part of the convection of the laser-melted pool. 

**Figure 8:**
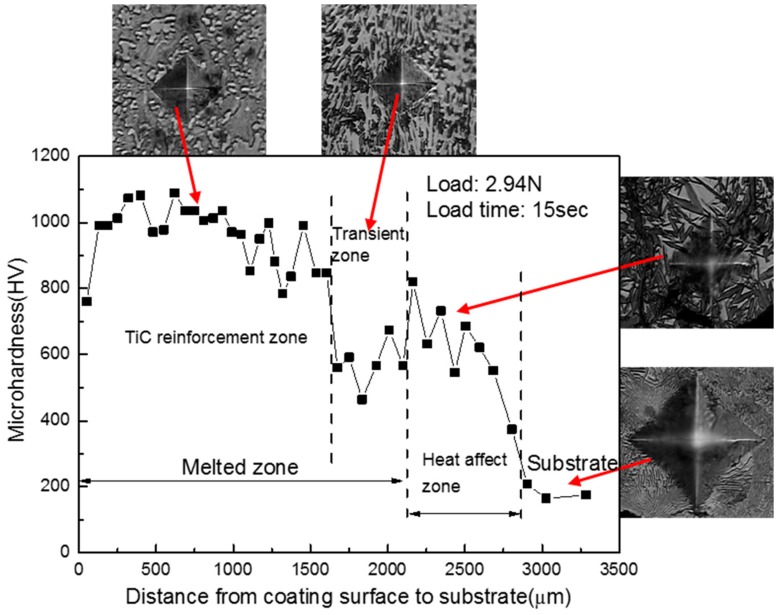
The microhardness distribution with the depth from the coating surface to the substrate in cross-section.

Figure 8 shows the microhardness distribution with the depth from the coating surface to the substrate in cross-section. The microhardness value on the coating was between 800 HV0.3 and 1100 HV0.3, the average microhardness value of the coating was up to 960 HV0.3. This was about five times that of the substrate. Obviously, the TiC phase and the two matrix phases were predominant for the microhardness of the coating. The microhardness of the transition zone was lower than the coating and the heat-affected zone. It was also closely related to its microstructure: there was only developed columnar crystal without TiC and martensite. The microhardness of the heat-affected zone gradually decreased from about 800 HV0.3 to the level of the substrate. This was in agreement with its microstructure characteristic, which changed from the quenching zone to the partial quenching zone. 

**Figure 9:**
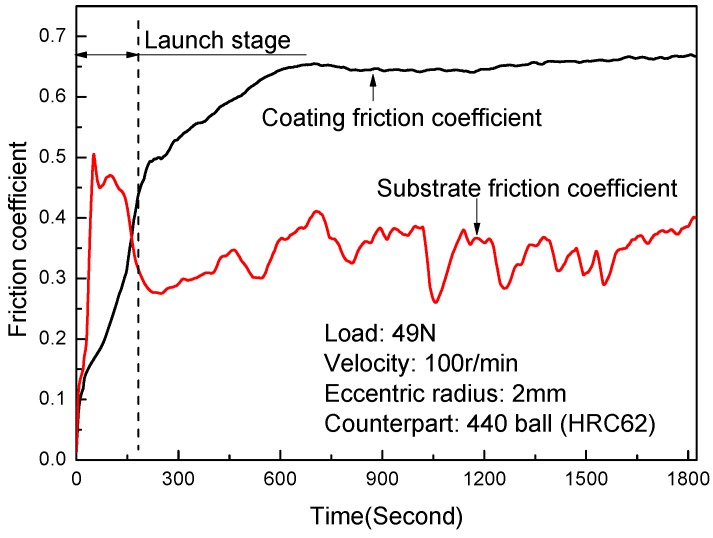
Friction coefficient curves of the coating and the substrate.

**Figure 10:**
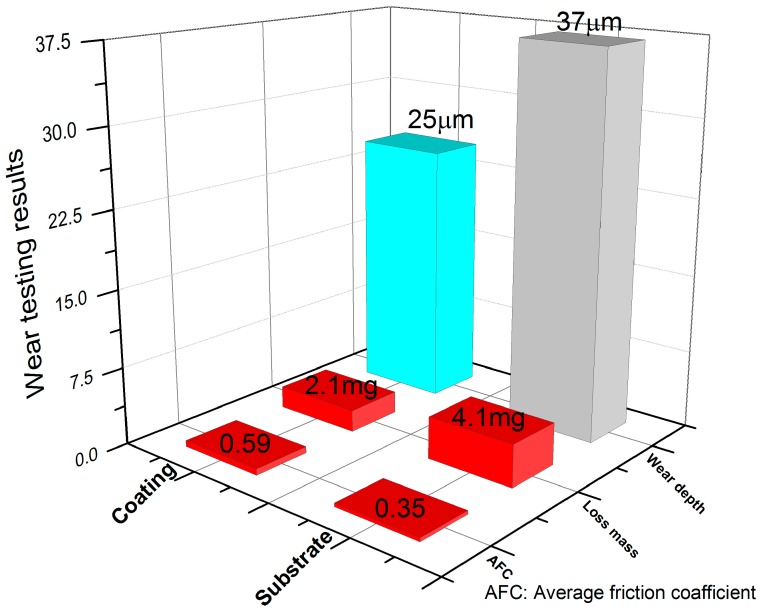
Wear testing results of the coating and the substrate.

Figure 9 shows friction coefficient curves of the coating and the substrate. Figure 10 shows the wear test results of the coating and the substrate. The wear test results revealed that the wear resistance of the composite coating was significantly improved relative to the substrate. Though the friction coefficient of the composite coating was higher than the substrate with the graphite phase in Figure 9, there was a smaller loss mass and a smaller wear depth of the composite coating in Figure 10. This agreed with the finding of Yan et al. [30]. The loss mass of the substrate was about two times that of the composite coating, and the wear depth of the substrate was about 1.5 times that of the composite coating.

The microhardness of the stainless steel ball counterpart was about 1050 HV0.3 under the same testing conditions of the coatings. It was about equal to the microhardness of the coatings, but five times higher than the substrate. This meant that the debris between the ball and the substrate disk came mainly from the cast iron with graphite, which had a good lubrication. However, the debris between the ball and the coatings disk came from both the coatings and the ball. This indicated that the debris between the ball and the coatings disk were not lubricated. The differences between both the debris and the microstructure were the main reason for the difference of the friction coefficient between the composite coating and the substrate in Figure 9 [30]. In addition, it was clear that the microhardness difference among the stainless steel ball and the coatings and the substrate was the main reason for the mass loss and wear depth in Figure 10.

**Figure 11:**
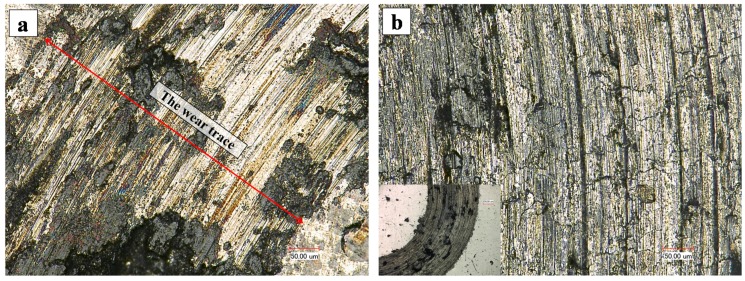
Optical micrographs of the wear trace: (**a**) coating; and (**b**) substrate.

Figure 11 shows the SEM images of the wear surface of both the coating and the substrate. Figure 11a clearly shows that the abraded coatings surface is characterized by fine and shallower grooves, which formed as the debris and the ball counterpart surface plough across the surface, removing or pushing material into ridges along the sides of the grooves [31]. Then, the removed material became the debris and exacerbated the wear between the coatings and the ball. Figure 11b exhibits that the wear tracks of the substrate was relatively smooth with a few long and deep grooves. The wear surface of the substrate was contaminated by the graphite indicating that the graphite in the substrate was in action as a lubricant during the wear testing.

## 4. Conclusions

The TiC-reinforced Fe-based composite coating was produced on the surface of a pearlite matrix grey iron using Ti powder by laser cladding. The TiC-reinforced phase was uniformly distributed in the coating. The coating matrix was composed of two kinds of *α*-Fe phases. The microstructure characteristic caused the microhardness of the coating to rise to about 1000 HV. Compared with the coating and the heat-affected zone, the developed columnar crystal without TiC and martensite in the transition zone were the main reasons for the lowest microhardness value. Since the graphite phase was replaced by the TiC phase during laser cladding, both the wear resistance of the composite coating and the friction coefficient rose significantly. The research results indicated that the grey cast iron could be strengthened or reproduced by laser cladding with the appropriate process.

## Figures and Tables

**Table 1:** EDS analysis data of the different phases in Figure 5.

Test Point	Fe (at %)	Ti (at %)	Si (at %)	C (at %)	Matrix
I (Light black particles phase)	5.09	91.16	–	3.75	ZAF
II (Grey matrix phase)	85.99	7.44	3.77	2.80	ZAF
III (Grey-white matrix phase)	93.71	2.93	–	3.36	ZAF

**Table 2:** EDS analysis data of the different parts in the laser cladding coating.

Test Point	Fe (at %)	Ti (at %)	Si (at %)	C (at %)	Matrix
Top of the laser cladding zone	73.67	20.55	3.23	2.55	ZAF
Middle of the laser cladding zone	76.17	18.52	3.09	2.22	ZAF
Bottom of the laser cladding zone	84.73	9.79	3.45	2.02	ZAF

## References

[1] Hütter G., Zybell L., Kuna M. (2015). Micromechanisms of fracture in nodular cast iron: From experimental findings towards modeling strategies—A review. Eng. Fract. Mech..

[2] Chen J., Xue L., Wang S.-H. (2011). Experimental studies on process-induced morphological characteristics of macro- and microstructures in laser consolidated alloys. J. Mater. Sci..

[3] Mellouli D., Haddar N., Köster A., Toure A.M.-L. (2011). Thermal fatigue of cast irons for automotive application. Mater. Des..

[4] Sun J.Q., Yan Z.F., Cui H.Z., Li J., Wang J.S., Chen Y.B. (2010). Surface catalysis gaseous nitriding of alloy cast iron at lower temperature. Catal. Today.

[5] Beyhaghi M., Kiani-Rashid A.R., Kashefi M., Khaki J.V., Jonsson S. (2015). Effect of powder reactivity on fabrication and properties of NiAl/Al_2_O_3_ composite coated on cast iron using spark plasma sintering. Appl. Surf. Sci..

[6] Fernandes F., Cavaleiro A., Loureiro A. (2012). Oxidation behavior of Ni-based coatings deposited by PTA on gray cast iron. Surf. Coat. Technol..

[7] Yang X., Zhang Z., Wang J., Ren L. (2015). Investigation of nanomechanical properties and thermal fatigue resistance of gray cast iron processed by laser alloying. J. Alloys Compd..

[8] Pang Z., Zhou H., Zhang P., Cong D., Meng C., Wang C., Ren L. (2015). Study on quantitative relation between characteristics of striature bionic coupling unit and wear resistance of gray cast iron. Appl. Surf. Sci..

[9] Jing Z., Zhou H., Zhang P., Wang C., Meng C., Cong D. (2013). Effect of thermal fatigue on the wear resistance of graphite cast iron with bionic units processed by laser cladding WC. Appl. Surf. Sci..

[10] Majumdar J.D., Manna I. (2011). Laser material processing. Int. Mater. Rev..

[11] Chen J., Wang S.-H., Xue L. (2012). On the development of microstructures and residual stresses during laser cladding and post-heat treatments. J. Mater. Sci..

[12] Chen Z.-K., Zhou T., Zhang H.-F., Yang W.-S., Zhou H. (2015). Influence of Orientations of Bionic Unit Fabricated by Laser Remelting on Fatigue Wear Resistance of Gray Cast Iron. J. Mater. Eng. Perform..

[13] Liu Y., Zhou H., Yang C.Y., Cheng J.Y. (2015). Thermal fatigue resistance of bionic compacted graphite cast iron treated with the twice laser process in water. Strength Mater..

[14] Bathe R., Krishna V.S., Nikumb S.K., Padmanabham G. (2014). Laser surface texturing of gray cast iron for improving tribological behavior. Appl. Phys. A.

[15] Duarte M., Lasagni A., Giovanelli R., Narciso J., Louis E., Mücklich F. (2008). Increasing lubricant lifetime by grooving periodical patterns using laser interference metallurgy. Adv. Eng. Mater..

[16] Walsh F.C., de Leon C.P. (2014). A review of the electrodeposition of metal matrix composite coatings by inclusion of particles in a metal layer: An established and diversifying technology. Trans. Inst. Met. Finish..

[17] Gallo S.C., Alam N., O’Donnell R. (2013). In-situ precipitation of TiC upon PTA hardfacing with grey cast iron and titanium for enhanced wear resistance. Surf. Coat. Technol..

[18] Gopagoni S., Hwang J.Y., Singh A.R.P., Mensah B.A., Bunce N., Tiley J., Scharf T.W., Banerjee R. (2011). Microstructural evolution in laser deposited nickel-titanium-carbon in situ metal matrix composites. J. Alloys Compd..

[19] Lekatou A., Karantzalis A.E., Evangelou A., Gousia V., Kaptay G., Gacsi Z., Baumli P., Simon A. (2015). Aluminium reinforced by WC and TiC nanoparticles (ex-situ) and aluminide particles (in-situ): Microstructure, wear and corrosion behavior. Mater. Des..

[20] Grum J., Šturm R. (2004). A new experimental technique for measuring strain and residual stresses during a laser remelting process. J. Mater. Process. Technol..

[21] Karamış M.B., Yıldızlı K. (2010). Surface modification of nodular cast iron: A comparative study on graphite elimination. Mater. Sci. Eng. A.

[22] Sun G., Zhou R., Li P., Feng A., Zhang Y. (2011). Laser surface alloying of C-B-W-Cr powders on nodular cast iron rolls. Surf. Coat. Technol..

[23] Zhu L.-N., Xu B.-S., Wang H.-D., Wang C.-B. (2012). Microstructure and nanoindentation measurement of residual stress in Fe-based coating by laser cladding. J. Mater. Sci..

[24] Molian P.A. (1982). Effect of fusion zone shape on the composition uniformity of laser surface alloyed iron. Scr. Metall..

[25] Kou S., Wang Y.H. (1986). Three-dimension convection in laser melted pools. Metall. Trans. A.

[26] Limmaneevichitr C., Kou S. (2000). Experiments to simulate effect of Marangoni convection on weld pool shape. Weld. Res. Suppl..

[27] Drezet J.-M., Pellerin S., Bezencon C., Mokadem S. (2004). Modelling the Marangoni convection in laser heat treatment. J. Phys. IV France.

[28] Abboud J.H. (2012). Microstructure and erosion characteristic of nodular cast iron surface modified by tungsten inert gas. Mater. Des..

[29] Alabeedi K.F., Abboud J.H., Benyounis K.Y. (2009). Microstructure and erosion resistance enhancement of nodular cast iron by laser melting. Wear.

[30] Yan H., Wang A., Xiong Z., Xu K., Huang Z. (2010). Microstructure and wear resistance of composite layers on a ductile iron with multicarbide by laser surface alloying. Appl. Surf. Sci..

[31] Garcia-Cordovill C., Narciso J., Louis E. (1996). Abrasive wear resistence of aluminium alloy/ceramic particulate composites. Wear.

